# A computer-aided diagnosing system in the evaluation of thyroid nodules—experience in a specialized thyroid center

**DOI:** 10.1186/s12957-019-1752-z

**Published:** 2019-12-06

**Authors:** Shujun Xia, Jiejie Yao, Wei Zhou, Yijie Dong, Shangyan Xu, Jianqiao Zhou, Weiwei Zhan

**Affiliations:** Department of Ultrasound, Rui Jin Hospital, Shanghai Jiao Tong University School of Medicine, 197 Rui Jin Er Road, Huang Pu District, Shanghai, 200025 People’s Republic of China

**Keywords:** Thyroid nodule, CADs, Experienced radiologists

## Abstract

**Background:**

The evaluation of thyroid nodules with ultrasonography has created a large burden for radiologists. Artificial intelligence technology has been rapidly developed in recent years to reduce the cost of labor and improve the differentiation of thyroid malignancies. This study aimed to investigate the diagnostic performance of a novel computer-aided diagnosing system (CADs: S-detect) for the ultrasound (US) interpretation of thyroid nodule subtypes in a specialized thyroid center.

**Methods:**

Our study prospectively included 180 thyroid nodules that underwent ultrasound interpretation. The CADs and radiologist assessed all nodules. The ultrasonographic features of different subtypes were analyzed, and the diagnostic performances of the CADs and radiologist were compared.

**Results:**

There were seven subtypes of thyroid nodules, among which papillary thyroid cancer (PTC) accounted for 50.6% and follicular thyroid carcinoma (FTC) accounted for 2.2%. Among all thyroid nodules, the CADs presented a higher sensitivity and lower specificity than the radiologist (90.5% vs 81.1%; 41.2% vs 83.5%); the radiologist had a higher accuracy than the CADs (82.2% vs 67.2%) for diagnosing malignant thyroid nodules. The accuracy of the CADs was not as good as that of the radiologist in diagnosing PTCs (70.9% vs 82.1%). The CADs and radiologist presented accuracies of 43.8% and 60.9% in identifying FTCs, respectively.

**Conclusions:**

The ultrasound CADs presented a higher sensitivity for identifying malignant thyroid nodules than experienced radiologists. The CADs was not as good as experienced radiologists in a specialized thyroid center in identifying PTCs. Radiologists maintained a higher specificity than the CADs for FTC detection.

## Background

The incidence of thyroid cancer has increased exponentially in the past decades and is ascribed to the improved ultrasonographic techniques and the application of fine-needle aspiration (FNA) [1, 2]. Among these new cases, papillary thyroid cancer (PTC) accounts for the largest percentage with a high number of papillary thyroid microcarcinomas (PTMCs, < 1 cm) [3]. However, the overall mortality of thyroid cancer has remained stable during these years [2]. The growing incidence of thyroid nodules also causes an increased burden to radiologists in diagnosing thyroid cancers based on ultrasound (US) imaging, which outperforms other imaging modalities in diagnosing thyroid nodules.

Artificial intelligence (AI) technology has been developed in recent years. Preliminary studies have shown major AI applications in imaging the breast [4], fetus [5], carotid [6], thyroid [7], liver [8], etc. Several deep learning technologies for the ultrasound computer-aided diagnosis system (CADs) are frequently utilized in investigations to support the clinical diagnosis [9]. A new CADs for thyroid ultrasound imaging, also known as “S-detect,” has been recently introduced to improve the thyroid US interpretation and provide assistance in the morphologic analysis of thyroid nodules. The proposed CADs provides preprocessing and refines segmentation processing before the ultrasonographical features are extracted from thyroid ultrasound images. The automatic classification system was developed after reviewing the extracted ultrasonographic features and validating the performance by comparing the results to the pathological results.

The CADs was designed with the purpose of increasing the diagnostic confidence for accurate and consistent recommendations. S-detection was preliminarily tested in a small number of patients [10]. In the current study, we extended the sample volume and aimed to investigate the diagnostic performance of this novel CADs in predicting thyroid nodules. This is the first report of the CADs evaluating pathological subtypes of thyroid nodules. Moreover, we compare the abilities of the CADs and experienced radiologists in a specialized thyroid center for thyroid US interpretation.

## Methods

### Patients

A total of 180 thyroid lesions in 171 consecutive patients who were scheduled for US-guided FNA or US examinations prior to scheduled surgery in our department from June 12, 2017,~June 30, 2017, were enrolled in this study. None of the patients had previously undergone surgeries in the neck. There were 32 male and 139 female patients, and the mean age was 47.2 years (range 21–83 years). This prospective study was approved by the Institutional Review Board of our hospital, and written informed consent was acquired from patients before the US examination.

Pathological analysis was performed to diagnose suspected nodules that underwent surgery. Benign nodules were diagnosed based on any of the following criteria: (1) benign according to FNA cytology or postoperative pathology or (2) benign US features including purely cystic or partially cystic nodules with comet tail artifacts or a spongiform appearance.

### US examination

**Fig. 1:**
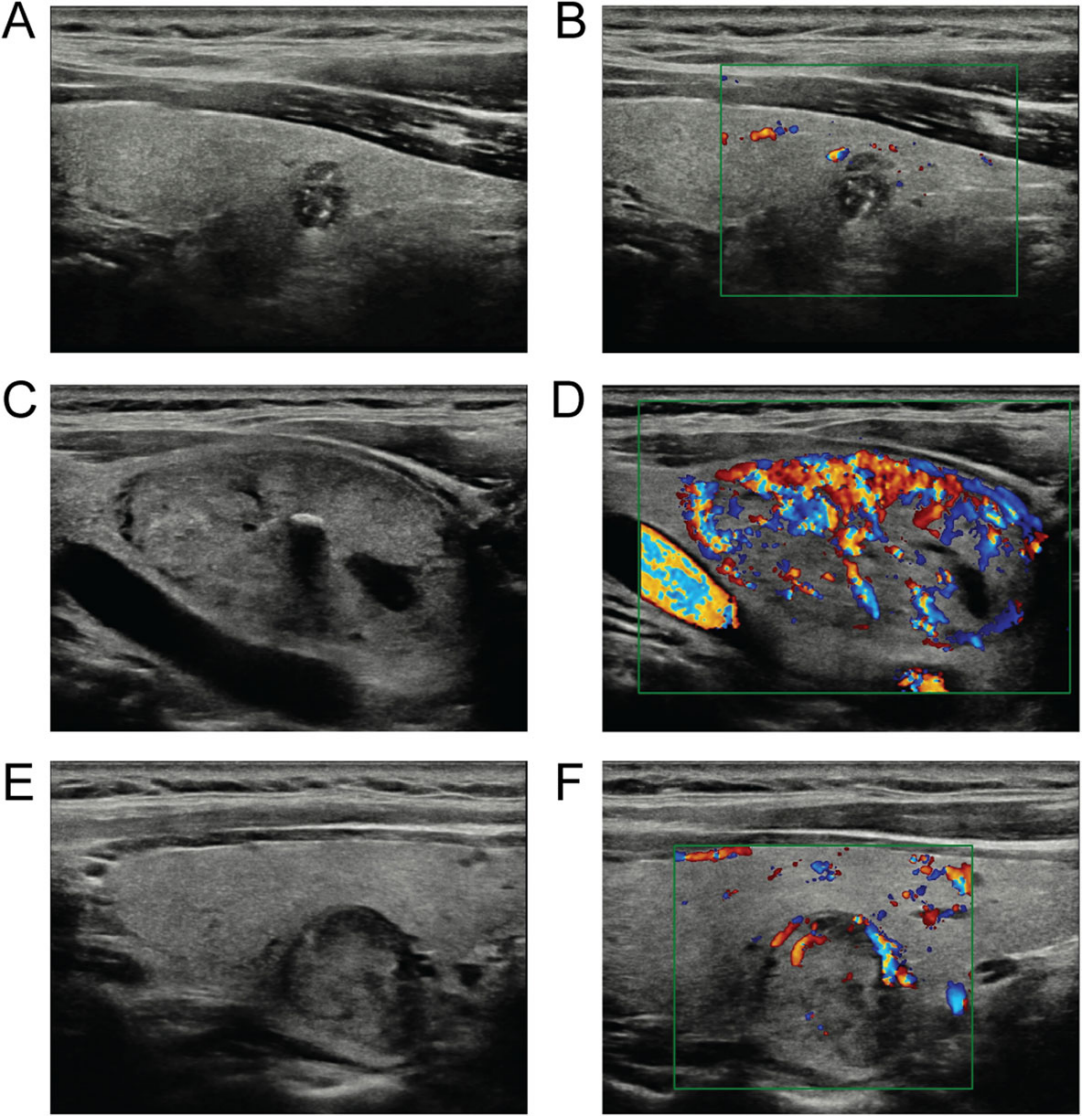
Grayscale and Doppler US examination of thyroid nodules. **a**, **b** A solid hypoechoic nodule with microcalcifications, a nonparallel pattern and low blood flow; **c**, **d** A well-defined heterogeneous nodule with macrocalcifications, a parallel pattern and high blood flow; **e**, **f** An ovoid to round hypoechoic nodule with low blood flow

RS80A with Prestige (Samsung Medison, Co., Ltd., Seoul, Korea) ultrasound diagnostic equipment was applied for the thyroid image acquisition with a 3–12 MHz linear array probe. The US features of the thyroid nodules were analyzed by one experienced radiologist with 20 years of experience in US thyroid examinations. The clinical history of the patients, including the results of prior US examinations and blood tests, was presented to the radiologist before imaging. The patients were placed in a supine position, and the anterior neck area was fully exposed. Dynamic scanning from the superior of the thyroid lobe to the inferior of the thyroid lobe and from the lateral neck to trachea was performed. Static images of the transverse and longitudinal dimensions of each target thyroid nodule were routinely collected. The grayscale ultrasonographic features of the thyroid nodules were observed, including composition (solid: no obvious cystic content, mainly solid: < 50% cystic, mainly cystic: >  50% cystic or spongiform appearance), echogenicity, margin (well-defined, lobulated, or ill-defined), calcification (microcalcifications, macrocalcifications, eggshell calcifications, mixed calcifications that contain both micro- and macrocalcifications, none), orientation, and shape. Color Doppler ultrasound was also included to analyze the degree of blood flow in the suspected thyroid nodule. We divided the blood flow according to a previous report [11], as follows: grade 0, without blood flow; grade I, low blood flow with 1–2 punctuate or rod-like blood vessels; grade II, medium blood flow with three or four blood vessels, one of which is longer than the radius of the nodule; and grade III, high blood flow with more than four visible blood vessels or interconnected angiogenesis pattern, interwoven into a network. (Fig. 1).

### Application of the computer-aided diagnosing system

**Fig. 2:**
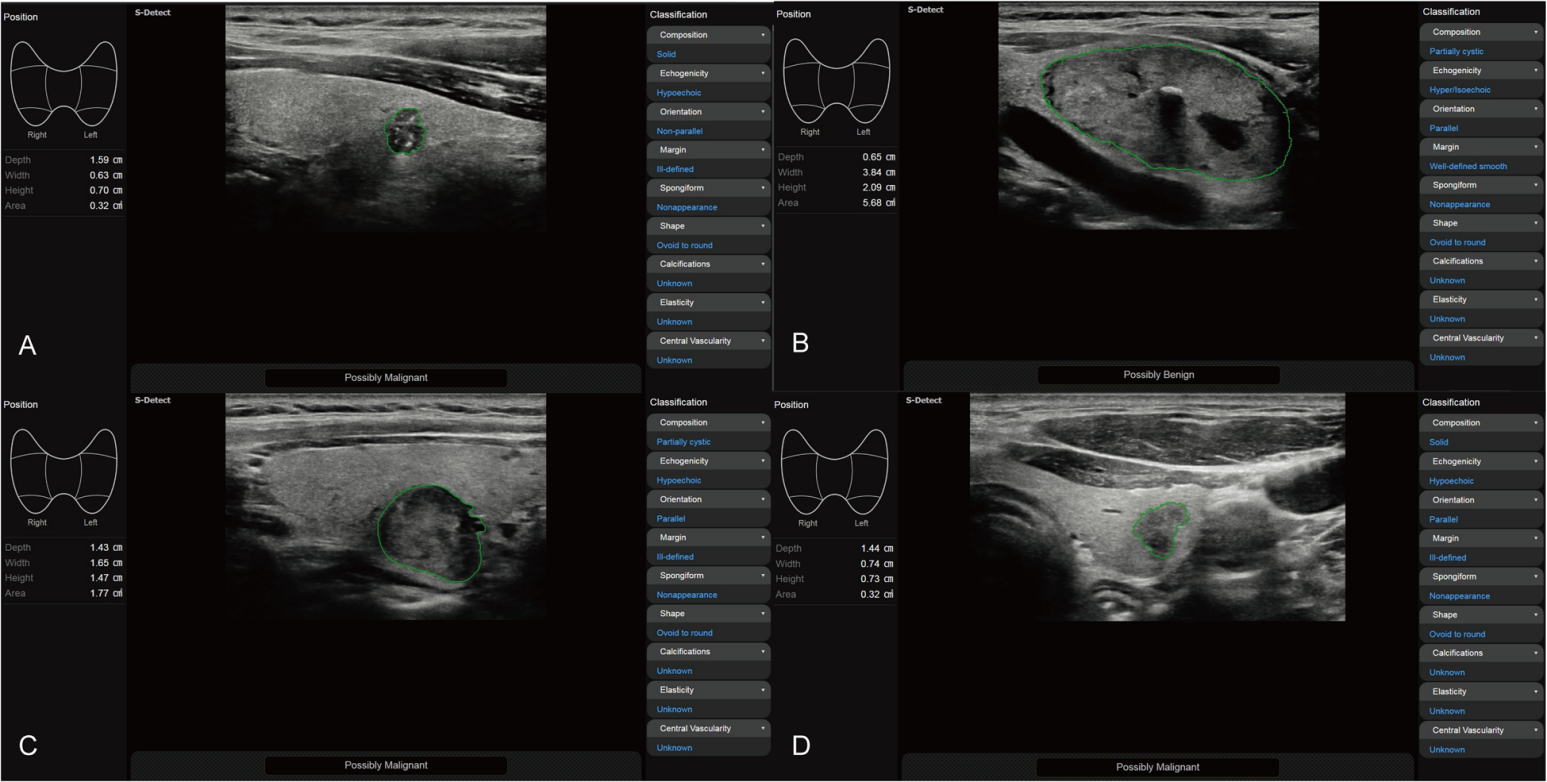
Application of the CADs on thyroid nodules. **a** A PTC nodule was identified as possibly malignant with the CADs; **b** A follicular thyroid adenoma was identified as possibly benign with the CADs; **c** A follicular thyroid carcinoma was identified as possibly malignant with the CADs. **d** Subacute thyroiditis was identified as possibly malignant with the CADs

Real-time CAD system software (S-Detect for Thyroid; Samsung Medison, Co. Ltd., Seoul, Korea) using artificial intelligence was integrated into the RS80A with Prestige (Samsung Medison, Co. Ltd., Seoul, Korea) US diagnostic system. The US features of the thyroid nodules identified by the CADs were reviewed by another senior radiologist who had 20 years of experience in diagnostic thyroid imaging. When the CADs was applied, a region of interest (ROI) was automatically drawn along the border of the target nodule by the US unit, and several candidates were available for the selection (Fig. 2). When the boundary of the nodule that was automatically drawn by the CADs was considered insufficient for the evaluation, a manual drawing of the boundary was applied. The US features, including composition, echogenicity, orientation, margin, spongiform appearance, shape, calcifications, and vascularity, were used for the analysis. The final evaluations from the CADs were in dichotomized form as possibly benign or possibly malignant.

### Statistical analysis

All tests and calculations were performed with Statistical Program for Social Sciences 19.0 software (SPSS, Chicago, IL, USA). Descriptive variables are presented as the mean ± SD. Categorical variables are described as proportions. Chi-square (*X*^2^) tests and Fisher’s exact tests were applied to compare the categorical variables. An independent two-sample *t* test was used to compare the nodule sizes between two independent groups. ROC curves and AUCs were used to compare the diagnostic efficiencies of the CADs and the radiologists. The sensitivity, specificity, positive predictive value (PPV), negative predictive value (NPV), and diagnostic accuracy were calculated with diagnostic testing. Cohen’s kappa coefficient was used to analyze the interobserver agreement for each US feature of the thyroid nodules. *P* < 0.05 was considered statistically significant.

## Results

Of these 180 nodules, the malignant thyroid nodules were papillary thyroid carcinomas (PTCs, *n* = 91) and follicular thyroid carcinomas (FTCs, *n* = 4), while the benign thyroid nodules were follicular thyroid adenomas (FTAs, *n* = 25), goiters (*n* = 46), cysts (*n* = 10), subacute thyroiditis (*n* = 2), and Hashimoto’s thyroiditis (*n* = 2). In total, 75 benign nodules were confirmed with FNA or a postoperative pathological diagnosis, and 10 were confirmed with benign US features. PTCs accounted for 50.6% of all thyroid nodules, and FTCs accounted for 2.2% of all thyroid nodules. There were 16.1% (*n* = 29) follicular thyroid neoplasms, including FTAs and FTCs. Table 1 summarizes the grayscale and color Doppler ultrasonographic features of the thyroid nodules based on different subtypes. Solid nodules with hypoechogenicity accounted for the largest proportion among the 180 lesions. The follicular neoplasms were significantly larger than PTCs and benign lesions, such as goiters, cysts, and thyroiditis (*P* = 0.001), whereas the FTAs were almost the same size as FTCs. Ill-defined margins were more likely to be found in PTCs (*P* = 0.000) than in follicular neoplasms and were often observed in thyroiditis than in other benign neoplasms; nevertheless, well-defined margins were more frequently observed in follicular neoplasms, goiters, and cysts than in other subtypes. The four FTCs were all found to have ill-defined margins. Nonparallel patterns were more often observed in PTCs than in the other subtypes (*P* = 0.002). Additionally, follicular neoplasms were more likely to have an ovoid to round shape (*P* = 0.000) and medium to high blood flow (*P* = 0.007) than PTCs in the current study. There were no significant differences regarding composition and calcifications among all the thyroid nodule subtypes.

**Table 1:** US features of thyroid nodules based on pathological subtypes

US features		Pathological subtypes					*P* value
	PTC ^*$^	FTC^*#^	FTA^*#^	Goiter	Cyst	Thyroiditis	
Size (Mean ± SD, mm)	P = 0.001^*^				P = 0.003^*#*^		0.003
	9.41 ± 6.98	15.56 ± 4.76	14.61 ± 8.86	9.68 ± 6.65	11.03 ± 6.46	5.30 ± 2.49	
Composition							0.280
Solid	90 (98.9%)	4 (100.0%)	24 (96.0%)	45 (97.8%)	0 (0.0%)	4 (100.0%)	
Mainly solid	1 (1.1%)	0 (0.0%)	1 (4.0%)	1 (2.2%)	4 (40.0%)	0 (0.0%)	
Mainly cyst	0 (0.0%)	0 (0.0%)	0 (0.0%)	0 (0.0%)	5 (50.0%)	0 (0.0%)	
Spongiform	0 (0.0%)	0 (0.0%)	0 (0.0%)	0 (0.0%)	1 (10.0%)	0 (0.0%)	
Echogenicity					P = 0.018^*$*^		0.038
Hyper	2 (2.2%)	0 (0.0%)	2 (8.0%)	0 (0.0%)	0 (0.0%)	0 (0.0%)	
Iso	5 (5.5%)	0 (0.0%)	7 (28.0%)	9 (19.6%)	0 (0.0%)	1 (25.0%)	
Hypo	60 (65.9%)	3 (75.0%)	12 (48.0%)	31 (67.4%)	0 (0.0%)	3 (75.0%)	
Markedly	4 (4.4%)	1 (25.0%)	0 (0.0%)	0 (0.0%)	0 (0.0%)	0 (0.0%)	
Anechoic	0 (0.0%)	0 (0.0%)	0 (0.0%)	0 (0.0%)	10 (100.0%)	0 (0.0%)	
Heterogenous	20 (22.0%)	0 (0.0%)	4 (16.0%)	6 (13.0%)	0 (0.0%)	0 (0.0%)	
Margin	P = 0.000^*^				P = 0.002^*$*^		0.168
Well-defined	5 (5.5%)	0 (0.0%)	13 (52.0%)	37 (80.4%)	10 (100.0%)	0 (0.0%)	
Lobulated	26 (28.6%)	0 (0.0%)	3 (12.0%)	2 (4.4%)	0 (0.0%)	0 (0.0%)	
Ill-defined	60 (65.9%)	4 (100.0%)	9 (36.0%)	7 (15.2%)	0 (0.0%)	4 (100.0%)	
Calcification							0.277
Micro	52 (57.1%)	0 (0.0%)	8 (32.0%)	17 (37.0%)	6 (60.0%)	0 (0.0%)	
Macro	7 (7.7%)	2 (50.0%)	0 (0.0%)	7 (15.2%)	0 (0.0%)	1 (25.0%)	
Eggshell	2 (2.2%)	0 (0.0%)	1 (4.0%)	3 (6.5%)	1 (10.0%)	0 (0.0%)	
Mixed	4 (4.4%)	0 (0.0%)	3 (12.0%)	2 (4.3%)	0 (0.0%)	0 (0.0%)	
None	26 (28.6%)	2 (50.0%)	13 (52.0%)	17 (37.0%)	3 (30.0%)	3 (75.0%)	
Orientation	P = 0.002^*^				P = 0.000^*$*^		0.004
Parallel	50 (54.9%)	4 (100.0%)	21 (84.0%)	39 (84.8%)	7 (70.0%)	4 (100.0%)	
Nonparallel	41 (45.1%)	0 (0.0%)	4 (16.0%)	7 (15.2%)	3 (30.0%)	0 (0.0%)	
Shape	P = 0.000^*^				P = 0.001^*$*^		0.044
Ovoid to round	12 (13.2%)	0 (0.0%)	15 (60.0%)	16 (34.8%)	10 (100.0%)	0 (0.0%)	
Irregular	79 (86.8%)	4 (100.0%)	10 (40.0%)	30 (65.2%)	0 (0.0%)	4 (100.0%)	
Blood flow	P = 0.007^*^						0.852
Without	0 (0.0%)	0 (0.0%)	0 (0.0%)	0 (0.0%)	9 (90.0%)	0 (0.0%)	
Low	76 (83.5%)	2 (50.0%)	14 (56.0%)	32 (71.1%)	1 (10.0%)	3 (75.0%)	
Medium	10 (11.0%)	1 (25.0%)	7 (28.0%)	7 (15.6%)	0 (0.0%)	1 (25.0%)	
High	5 (5.5%)	1 (25.0%)	4 (16.0%)	6 (13.3%)	0 (0.0%)	0 (0.0%)	

*PTC* papillary thyroid carcinoma, *FTC* follicullar thyroid carcinoma, *FTA* follicullar thyroid adenoma, *US* ultrasound

^*^PTC vs FTC + FTA

^$^PTC vs goiter, cyst, and thyroiditis

^#^FTC + FTA vs goiter, cyst, and thyroiditis

Table 2 presents the assessments of the CADs and radiologist of the 180 thyroid nodules based on different subtypes. When evaluating PTCs, the CADs had a higher rate of malignancies than the radiologist (90.1% vs 83.5%). Among the follicular thyroid neoplasms, the CADs correctly diagnosed all four FTCs, while the radiologist found only one FTC; however, the radiologist correctly evaluated 23 (92.0%) FTAs, while the CADs diagnosed only 10 (40.0%).

**Table 2:** Assessment of thyroid nodules according to CADs and radiologist

	Pathological subtypes						*P* value
	PTC ^*^	FTC^*#^	FTA^*#^	Goiter	Cyst	Thyroiditis	
CADs	P = 0.040^*^				P = 0.020^*#*^		0.267
PB	9 (9.9%)	0 (0.0%)	10 (40.0%)	20 (43.5%)	4 (40.0%)	1 (25.0%)	
PM	82 (90.1%)	4 (100.0%)	15 (60.0%)	26 (56.5%)	6 (60.0%)	3 (75.0%)	
Radiologist	P = 0.010^*^				P = 0.241^*#*^		0.098
PB	15 (16.5%)	3 (75.0%)	23 (92.0%)	38 (82.6%)	10 (100.0%)	0 (0.0%)	
PM	76 (83.5%)	1 (25.0%)	2 (8.0%)	8 (17.4%)	0 (0.0%)	4 (100.0%)	

*PTC* papillary thyroid carcinoma, *FTC* follicullar thyroid carcinoma, *FTA* follicullar thyroid adenoma, *CADs* computer-aided diagnosis system, *PB* possibly benign, *PM* possibly malignant

^*^PTC vs FTC + FTA

^#^FTC + FTA vs goiter, cyst, and thyroiditis

**Fig. 3:**
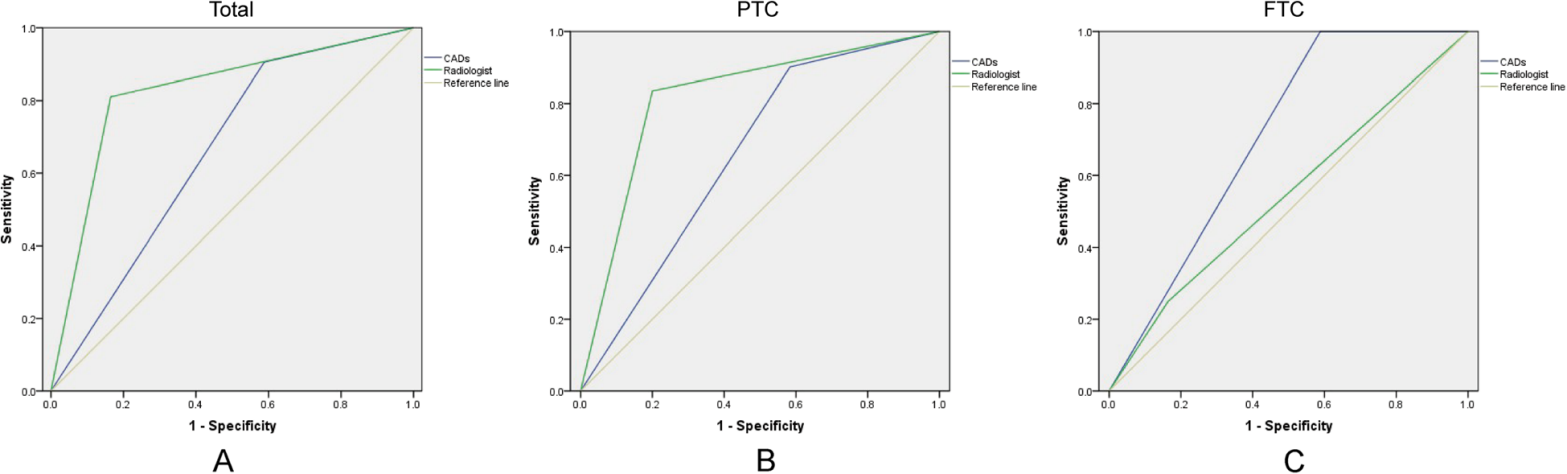
ROC curves showing the performance of the CADs and radiologist in evaluating malignant thyroid nodules. **a** Identifying the overall malignancies: Area under curve, CADs vs radiologist – 0.659 (0.577,0.740) vs 0.823 (0.758,0.887), *P* = 0.000 **b** Identifying PTCs: Area under curve, CADs vs radiologist – 0.659 (0.566,0.751) vs 0.818 (0.744,0.891), *P* = 0.000 **c** Identifying FTCs: Area under curve, CADs vs radiologist – 0.706 (0.523,0.889) vs 0.543 (0.240,0.846), *P* = 0.166

The diagnostic performance of the CADs and radiologist was compared and is shown in Table 3 and Fig. 3. When FTCs were excluded, the sensitivity of the CADs in diagnosing malignancies (PTC) was higher than that of the radiologist (90.1% vs 83.5%), while the specificity of the CADs in diagnosing malignancies (PTCs) was much lower than that of the radiologist (41.7% vs 80.0%); the accuracy of the CADs was not as good as that of the radiologist in diagnosing PTCs. When PTCs were excluded, the sensitivity of the CADs in diagnosing malignancies (FTCs) was higher than that of the radiologist (100.0% vs 25.0%), while the specificity of the CADs in diagnosing malignancies (FTC) was much lower than that of the radiologist (41.2% vs 83.5%). Both the CADs and radiologist had quite high NPVs (100.0% vs 95.9%) but quite low PPVs (7.4% vs 6.7%). When diagnosing FTCs, both the CADs and radiologist presented relatively low accuracies (43.8% vs 60.9%), and with no differences (*P* = 0.166). Among all of these thyroid nodules, the CADs presented a higher sensitivity and lower specificity than the radiologist (90.5% vs 81.1%; 41.2% vs 83.5%); however, the overall accuracy of the radiologist was higher than that of the CADs (82.2% vs 67.2%) for diagnosing malignant thyroid nodules.

**Table 3:** Performance of CADs and radiologist in evaluation of malignant thyroid nodules

	CADs					Radiologist				
	Sensitivity (%)	Specificity (%)	NPV (%)	PPV (%)	Accuracy (%)	Sensitivity (%)	Specificity (%)	NPV (%)	PPV (%)	Accuracy (%)
All	90.5	41.2	79.5	63.2	67.2	81.1	83.5	79.8	84.6	82.2
PTC	90.1	41.7	73.5	70.1	70.9	83.5	80.0	76.2	86.4	82.1
FTC	100.0	41.2	100.0	7.4	43.8	25.0	83.5	95.9	6.7	60.9

*NPV* negative predictive value, *PPV* positive predictive value, *PTC* papillary thyroid carcinoma, *FTC* follicullar thyroid carcinoma, *CADs* computer aided diagnosis system

The interobserver agreement between the CADs and radiologist in terms of US features was fair-to-substantial (kappa = 0.40~0.75): composition (kappa = 0.694), echogenicity (kappa = 0.742), orientation (kappa = 0.703), and spongiform appearance (kappa = 0.551). Poor interobserver agreement was considered “marginal,” as the kappa value was lower than 0.40 (kappa = 0.332). The extent of interobserver agreement between the CADs and radiologist in terms of the final evaluation was substantial (kappa = 0.719).

## Discussion

Various CADs options have become a hot research topic in the field of medical imaging as science and technology have developed. Various algorithms or software have been created to investigate the feasibility of applying a CADs to improve the detection of specific diseases [12, 13]. Ultrasound examinations are regarded as the first-line imaging method for the evaluation of thyroid nodules [14]. Our study is the first to evaluate a CADs for thyroid nodules according to different pathological subtypes. S-detect, as an automated imaging reporting system that was originally designed for breast cancer, was utilized in our center to study its ability to diagnose thyroid nodules and was compared with experienced radiologist in our center, which specializes in thyroids. This study showed that the sensitivity of the CADs in detecting PTCs was higher than that of the experienced radiologist. However, the CADs had a lower specificity and accuracy than the experienced radiologist in identifying PTCs. The CADs maintained a relatively lower performance than the experienced radiologist in identifying FTCs.

Sonographic patterns of thyroid cancers were established and published by the American Thyroid Association in 2015 to estimate the risk of malignancy and to guide FNAs [15]. The Thyroid Imaging Reporting and Data System (TI-RADS), a well-known quantitative risk stratification system for thyroid nodules, was also established based on the specific sonographic features of PTCs [16, 17]. The basic ultrasonographic features of evaluated thyroid nodules include composition, echogenicity, margin, calcifications, taller-than-wide appearance, shape, and blood flow, with different weights for each feature [18]. The most important aspect of these risk stratification systems is whether they offer a better diagnostic performance than the other systems and whether they are simple, which will provide easy implementation in everyday practice. The CADs in this study reviewed the images and extracted these basic ultrasonographic features, and the performance was validated according to the final pathological diagnosis. An intermediate suspicion (10–20%) of thyroid cancer is considered for solid and hypoechoic nodules. When irregular shapes, ill-defined margins, or taller than wide signs were present for the same nodule, a higher suspicion for malignancy is recommended [19]. The composition, echogenicity, orientation, margin, and shape were automatically analyzed by the CADs. Microcalcifications are also regarded as a suspected feature of thyroid malignancies [20, 21]. Vascularity could also be a complementary tool in the differentiation of thyroid nodules [22, 23]. Elasticity could be detected, but it was not included in the analysis at this time. As stiffness could be an indicator of aggressiveness [24], an evaluation of elasticity may improve the performance of the CADs in diagnosing thyroid nodules.

Follicular neoplasms presented a unique pattern of ultrasonographic characteristics. The follicular thyroid neoplasms were reported to be much larger in size than PTCs. Well-defined margins and regular shapes are commonly considered indications of benign thyroid nodules, but they are frequently observed in follicular adenomas and carcinomas [25], leading to an underestimation of the follicular thyroid nodule classifications. Our study also showed a different ultrasonographic pattern of follicular neoplasms from PTCs and goiters. This study demonstrated that follicular neoplasms were larger in size and more often ovoid or round in shape with well-defined margins than PTCs and goiters, which was consistent with previous studies [26]. Thus, follicular carcinoma, which can only be confirmed by histopathology, was highly likely to be considered benign under the TI-RADS risk stratification, which is a widely used categorization system in China. In our study, follicular neoplasms were more likely to be found with a larger size, ovoid shape, and well-defined margins, which are regarded as benign ultrasonographic features of thyroid nodules. In this study, only four FTCs were included; the CADs correctly diagnosed all four FTCs, while the radiologist found only one FTC. However, the radiologist correctly evaluated most of the FTAs (92.0%), while the CADs diagnosed no more than half of these lesions (40.0%). For follicular neoplasms, the CADs had a lower accuracy than the experienced radiologist in detecting FTCs, as radiologists maintained a higher specificity in identifying FTCs. However, there was a limited number of FTCs in our study, which may lead to bias. A larger sample size of FTCs could be included for future analyses.

Several studies have reported the performance of different types of CADs platforms for thyroid nodules, but most of these data were acquired only in the lab without clinical training. S-detect is a commercially used CADs; thus, it acquires data from patients. Our study showed that the performance of the CADs was not as good as that of the experienced radiologist in our center, which is a specialized center for thyroid ultrasound. The US CADs provided considerable sensitivity (> 0.0%), which was better than that of the experienced radiologist. Considering the labor required to interpret thyroid US images, using the CADs for a pre-evaluation could potentially save some time, with the radiologist making the final decision for the positive cases identified by the CADs. The specificity of the experienced radiologist was higher for identifying thyroid malignancies than that of the CADs. Thus, an experienced radiologist could rule out the benign thyroid nodules identified by the CADs. However, a comparison of the CADs and inexperienced radiologists in diagnosing thyroid nodules has not been conducted in this study.

There are still several limitations in this study. First, we included nodules subjected to US-guided FNAs or US examinations prior to scheduled surgery. Therefore, the proportion of malignancies was rather high, which may have influenced the diagnostic performance of the CADs. Second, non-mass lesions were not included in the study population since the analysis of the CADs was limited for non-mass lesions. Third, most of the malignancies were classical PTCs. As the US features of follicular variant PTCs, follicular thyroid carcinomas, medullary thyroid carcinomas, and other malignancies differ somewhat from those of classical PTCs, large population studies are required.

In conclusion, the ultrasound CADs (S-detect) presented a higher sensitivity in identifying malignant thyroid nodules than experienced radiologists. This study showed that the CADs was not as good as experienced radiologists in a specialized thyroid center in identifying PTCs. Radiologists maintained a higher specificity than the CADs for FTC detection.

## Data Availability

Not applicable.
